# Quality and Quantity of Rehabilitation Exercises Delivered By A 3-D Motion Controlled Camera: A Pilot Study

**DOI:** 10.4172/2329-9096.1000214

**Published:** 2014-07-29

**Authors:** Ravi Komatireddy, Anang Chokshi, Jeanna Basnett, Michael Casale, Daniel Goble, Tiffany Shubert

**Affiliations:** 1 Co-Founder and Chief Medical Officer, Reflexion Health, 3344 North Torrey Pines Ct, Suite 100, La Jolla, California, 92037, USA; 2 Lead PT and Clinical Accounts, Reflexion Health, 3344 North Torrey Pines Ct, Suite 100, La Jolla, California 92037, USA; 3 Clinical Affairs and People Manager, Reflexion Health, 3344 North Torrey Pines Ct, Suite 100, La Jolla, California 92037, USA; 4 Research Scientist, West Health Institute, 10350 North Torrey Pines Rd, La Jolla, California 92037, USA

**Keywords:** Rehabilitation, Virtual reality, Exercise, Therapy, Home exercise Program

## Abstract

**Introduction:**

Tele-rehabiliation technologies that track human motion could enable physical therapy in the home. To be effective, these systems need to collect critical metrics without PT supervision both in real time and in a store and forward capacity. The first step of this process is to determine if PTs (PTs) are able to accurately assess the quality and quantity of an exercise repetition captured by a tele-rehabilitation platform. The purpose of this pilot project was to determine the level of agreement of quality and quantity of an exercise delivered and assessed by the Virtual Exercise Rehabilitation Assistant (VERA), and seven PTs.

**Methods:**

Ten healthy subjects were instructed by a PT in how to perform four lower extremity exercises. Subjects then performed each exercises delivered by VERA which counted repetitions and quality. Seven PTs independently reviewed video of each subject’s session and assessed repetitions quality. The percent difference in total repetitions and analysis of the distribution of rating repetition quality was assessed between the VERA and PTs.

**Results:**

The VERA counted 426 repetitions across 10 subjects performing the four different exercises while the mean repetition count from the PT panel was 426.7 (SD = 0.8). The VERA underestimated the total repetitions performed by 0.16% (SD = 0.03%, 95% CI 0.12 – 0. 22). Chi square analysis across raters was χ^2^ = 63.17 (df = 6, p<.001), suggesting significant variance in at least one rater.

**Conclusion:**

The VERA count of repetitions was accurate in comparison to a seven member panel of PTs. For exercise quality the VERA was able to rate 426 exercise repetitions across 10 patients and four different exercises in a manner consistent with five out of seven experienced PTs.

## Introduction

Recovery from musculoskeletal trauma, stroke, and joint surgery is strongly correlated to total dose of exercise and therapy [1]. It is estimated the optimal dose of exercise to protect against a fall is a minimum of 50 hours [2]. Stroke patients have better outcomes when they receive a home exercise program for six to twelve months post-stroke, and those recovering from knee replacement demonstrate better out comes with greater total doses of therapy [1].

These research findings support greater patient engagement in the progress of their strength and endurance. However, systems to support patients in this endeavor are lacking. There are 24 million episodes of physical therapy care performed each year [3]. Over 90% of each episode of musculoskeletal physical therapy care is done by the patient in the home, typically as the “home exercise program” (HEP). The HEP is performed outside the purview of the physical therapist (PT). Aside from periodic visits with the PT to assess and progress, patients are expected to use non-interactive, low-quality paper handouts for guidance to perform a prescribed home exercise program and to track their own rehabilitation progress.

The majority of time spent during a physical therapy treatment is devoted to correcting a musculoskeletal impairment problem, allowing limited time for patient education. Not surprisingly, this system results in poor adherence and compliance rates with home exercise programs which are designed to maintain or improve the patient’s function. Researchers report adherence rates with HEPs of only 15–40% [4,5], contributing to prolonged recovery time, medical complications, and increased costs of care [6].

There is a lack of robust tools to observe the quality of home exercise performance outside of periodic in-clinic visits. PTs have little insight into patient progress through a home therapy plan. The inability to monitor performance in the home results in missed opportunities to provide corrective feedback, identify if a patient needs additional help, and provide motivation if necessary.

Telemedicine software applications for rehabilitation show potential to bridge this gap. Telemedicine can enhance the real time information provided to the patient on their progress and enable communication between providers and patients in the home [7]. Using the latest innovations in motion tracking sensors, originally designed for consumer video game consoles, tele-rehabilitation platforms have the potential to measure important physical therapy metrics related to patient motion. These metrics can be used by a PT to remotely assess patient progress and guide treatment in a more cost-effective, engaging, and efficient way [8]. Indeed, previous efforts aimed at using motion tracking systems associated with video game consoles such as the Nintendo^™^ Wii, and others, have shown promise in guiding patients through stroke and musculoskeletal rehabilitation in supervised, in-clinic environments [9,10].

To explore the clinical utility of commercial gaming systems as tele-rehabilitation platforms, the basic ability of the system to capture performance metrics should be demonstrated. Currently, self-report is the most common way to document progress outside of the clinic. However, tracking the number and quality of exercises may not be accurately reported by the patient to the PT.

Assessment of exercise quality, or “correctness”, is critical to promoting proper exercise form and timely recovery from injury. Exercise quality is typically evaluated during physical therapy as part of the plan of care. This may lack carryover into the real world setting. Also of concern is that adherence to poor exercise form may delay therapy-based healing and/or place patients at greater risk for re-injury [11,12].

The Virtual Exercise Rehabilitation Assistant, VERA, (Reflexion Health Inc., San Diego CA) is a tele-rehabiliation application that uses the Kinect^™^ motion tracking camera (Microsoft^®^ Inc., Redmond, WA) to guide a patient through a home exercise program without direct PT supervision. Using the Kinect^™^ camera and custom software the VERA is able to track the movement of over 20 joints simultaneously while guiding patients through a series of lower extremity exercises using an on-screen avatar. The VERA software automatically tracks patients’ exercise repetitions, and provides real-time, corrective feedback depending on whether repetitions are performed “correctly” according to pre-defined movement criteria. Repetition counts (i.e. quantity) and the number of optimal and sub-optimal repetitions (i.e. quality) are summarized for review by patients and PTs, either in real time or “store and forward” for later review.

The purpose of this pilot study was to determine the VERA’s accuracy for two clinical metrics, quantity and quality of an exercise repetition. These variables were calculated by examining 10 subjects performing repetitions of four different lower extremity exercises. The results from the VERA were subsequently compared to assessments from seven PTs who determined total repetition count and quality scores after reviewing videos of each subject’s exercise session. It was hypothesized that there would be acceptable agreement between VERA and the PTs.

## Methods

**Figure 1:**
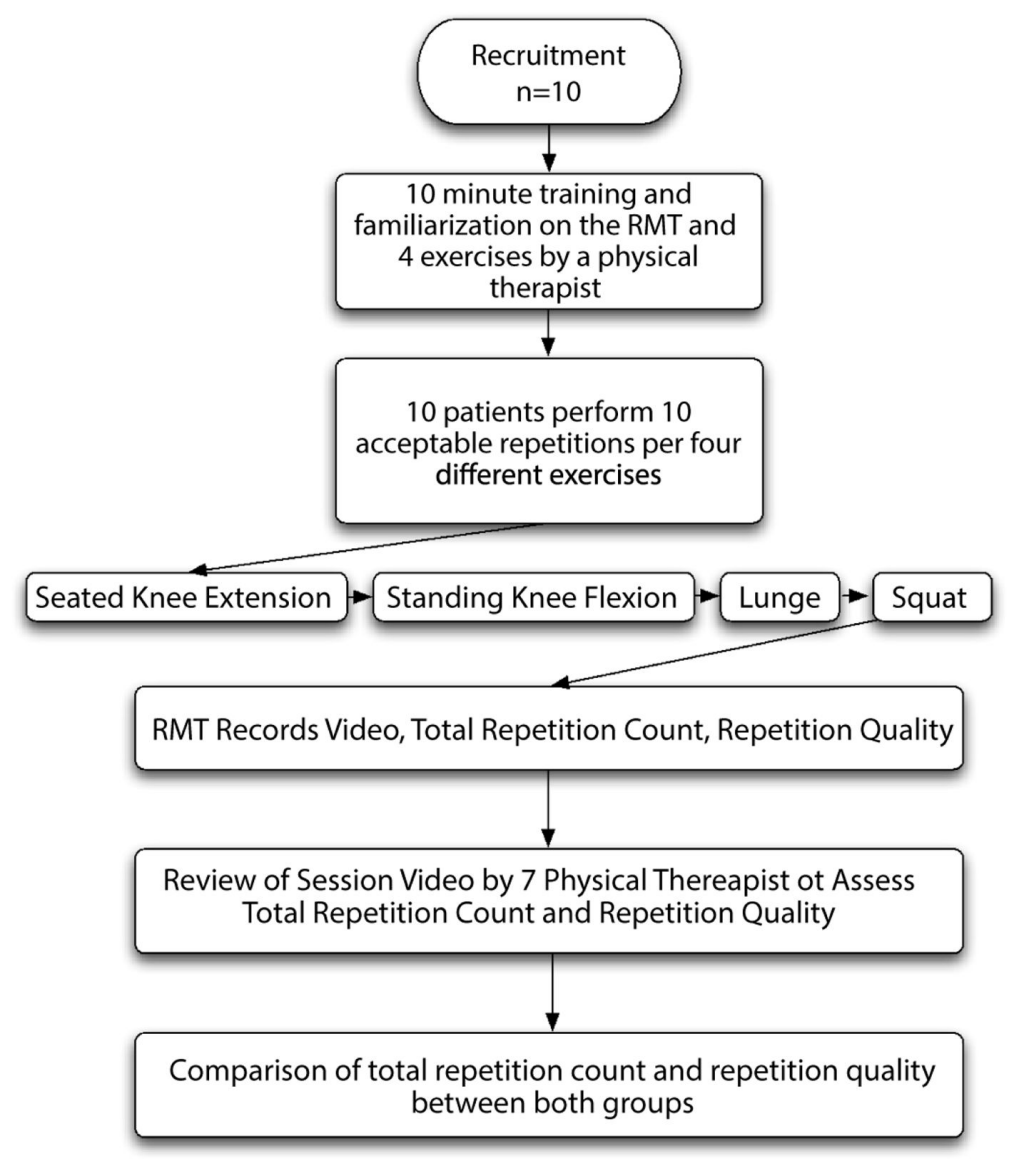
Diagram illustrating the study flow including the order and type of exercises performed.

Figure 1 illustrates the general study flow.

### Subjects

Ten healthy subjects age range (18–36) were recruited from the San Diego State University campus via flyer and word of mouth. Subjects contacted the study personnel who conducted an over the phone screen. Subjects were included that had no history of physical disability, lower extremity surgery, or limitation of lower extremity range of motion. This study was approved by the local Institutional Review Board. On the day of testing, each subject was brought onsite, the study was explained and they signed informed consent acknowledging they would be videotaped during the study.

Each participant was given a 10 minute training session on how to use the VERA (v1.0.77 Reflexion Health Inc. San Diego, CA). The training session included: how to perform the exercises optimally for camera detection, how to navigate the VERA, and interpretation of error messages displayed. Once subjects demonstrated independent ability to use the system they were instructed in the specific exercises.

A PT researcher provided instruction for four exercises - sitting knee extension, standing knee flexion, deep lunge, and squat in that order. These exercises were chosen for two reasons:

- They represent significant diversity in overall patterns of body configuration;
- Patients typically use additional objects such as chairs while performing these exercises and we wanted to determine if that would impact accuracy of measurement

The PT researcher demonstrated the exercises and then instructed the subject in how to perform the exercise. Once the subject demonstrated mastery of the exercise per the PT, then they were oriented to the VERA system.

### Orientation to VERA

A Windows 7 laptop computer, a Kinect^™^ camera, and a connection to a flat screen television that mirrored the laptop display were used to deploy the VERA software. The subject was oriented to the flat screen television where they could see an image of themselves. They were then instructed in how to follow the onscreen avatar and the image of themselves to perform the exercises. Subjects were oriented to corrective feedback provided by the VERA system. Once the subject indicated they were comfortable with system navigation and following exercise instruction, they started the first exercise.

### Exercise session

Upon completion of each exercise repetition the VERA recorded the repetition as “acceptable” or “unacceptable”. If a repetition was considered acceptable the onscreen repetition counter visible to the participant increased by one whole number. Examples of unacceptable repetitions included poor body positioning or sub-optimal exercise form as determined by the programmed parameters of the exercise. When a repetition was considered unacceptable the on-screen counter did not increase and the subject was given visual corrective feedback from the VERA. Examples of feedback include: 1) corrections of overall body position; and 2) correction of a specific body part.

The onscreen repetition counter only counted “acceptable” repetitions. However, all repetitions were marked for review by the therapists.

The session was simultaneously video recorded using standard RGB video by the Kinect^™^ camera. An exercise session was considered complete after patients achieved 10 acceptable repetitions for each of the four exercises as judged by the VERA.

### PT review

Given the inherent variability in PT assessment of exercise quality we chose a panel of seven PTs to observe retrospective videos of the participants and determine the total repetition count and assess of repetition quality [13]. Video review of physical therapy exercises has been previously shown to be an accurate and reliable method of exercise assessment [14]. Research suggests a minimum of seven raters provides both a large enough sample to get a proper representation of licensed PTs (varied levels of education, years of practice, specialization, etc.) as well as provide substantial power for tests of agreement [15].

PTs recruited held current licenses and had at least two years of clinical experience. Each PT worked in an outpatient setting and had experience instructing patients in the four selected exercises.

The PTs were scheduled for an observation session. Prior to observing the videos, they were provided a brief orientation to the study and signed informed consent.

To minimize bias, each PT was instructed to watch each subject perform 4 different exercises via video recording. The recordings of each subject were presented in a random order. Each PT watched each video recording twice.

During the first video review each PT was asked to count the number of repetitions from each subject for each of the four exercises. During the second video review each PT was asked to evaluate each repetition as “acceptable” or “unacceptable” in terms of repetition “correctness.” PTs recorded findings on a blank page. PTs were not given any additional information about the subjects (demographics, healthy, injured, etc.) and were blinded to VERA analysis. To mimic as much as possible the natural variability present in clinical setting, the PTs were not given any specific criteria a priori on acceptable or unacceptable parameters.

### Statistical data analysis

Data was analyzed using Matlab version R2011a (Mathworks, Natick MA)

### Total repetition count

The number of exercise attempts recorded by the VERA was compared to the number of attempts recorded by all PTs using a one-sided t-test with a Type 1 (alpha) error rate of 0.05. The 10 subjects were instructed to reach 10 acceptable repetitions for four different exercises for a target of at least 400 repetitions total across all subjects.

We defined an acceptable threshold for agreement between the VERA and PT panel as a 5% over or underestimation for counting total exercise repetitions across all subjects.

### Repetition quality

The quality of VERA repetitions was compared to the PTs. There was the potential for high variability in exercise quality among PT raters. This was difficult to predict prior to data analysis. To account for this variability, we first performed a Chi-square statistic with a type I error of 0.05 to compare results from the VERA and each PT. This approach was chosen because of the variance in evaluation of exercise form that exists in clinical practice.

The purpose was to assess if the frequency of categorical judgments could be treated the same among all raters, including the VERA. The Chi-square expected value was set as the average rating of acceptable or unacceptable repetitions across all repetitions for all seven PT raters. An a priori rejection of the null hypothesis across all raters would prompt a closer inspection of the data for PT outliers. The analysis would be repeated on the remaining raters and the VERA to assess consistency with regard to repetition quality.

If the null hypothesis was accepted, then all of the raters, including the VERA, agreed upon the parameters of the exercise. However, if the null hypothesis was rejected, all pairwise comparisons among the PTs would be performed to identify which raters had different metrics to evaluate an exercise.

### PT Inter-rater analysis

Upon completion of the data analysis to determine agreement between raters a pairwise analysis using a Chi square statistic was obtained for all raters including the VERA.

## Results

The 10 subjects were able to complete 10 acceptable repetitions, as determined by the VERA. Similarly, all seven PTs were able to review the video during two passes, as described above, to determine total repetition counts and assess repetition quality.

### Quantification of repetitions

The total repetition count for the PTs compared to VERA is shown in Table 1. The VERA counted 426 repetitions while PTs 1–6 counted 427 repetitions and PT 7 counted a total of 425 repetitions. Compared to each of the PTs the VERA underestimated the total repetition count on an average of 0.16% (SD = 0.03%, 95% CI 0.12 – 0.22). A one-sided t-test comparing both groups was significant (t (6) - = −72.05, p < 0.05), leading us to reject the null hypothesis that the VERA overestimated the repetition count of the PTs by more than 5%.

In Table 1, out of a total count of 426 repetitions across all subjects and exercises the VERA counted 400 as acceptable and 26 as unacceptable. PT 7 underestimated the repetition count compared to PTs 2–6 by two repetitions and by 1 repetition compared to PT1 and the VERA. PTs 1–7 exhibited a range of acceptable repetitions, from 374 to 423 with a mean of 403 and SD = 15. The PT assessment of unacceptable repetitions ranged from 2 to 53 with a mean of 24 and SD of 16.

### Rating movement quality

**Figure 2:**
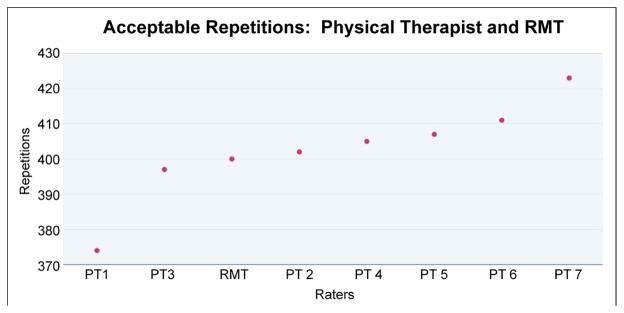
Scatter plot of acceptable exercise repetitions by rater.

The initial chi square test for independence performed for all raters, including the VERA, was = 63.17 (df = 6, p<.001). This result suggested at least one of the raters incorporated different metrics to evaluate quality. A pair-wise chi square (Table 2) and scatter plot of acceptable repetitions across all raters was reviewed (figure 2). This data revealed that PT1 and PT7 rated exercise repetitions inconsistent with the VERA as well as PTs 2–6.

## Discussion

In this study we compared the results between a potential tele-rehabiliation tool using the Kinect^™^ motion tracking camera to a group of PTs assessing exercise repetitions and quality for ten healthy volunteers performing 4 different lower extremity exercises.

The results suggests the VERA’s ability to assess the number of repetitions and the acceptability of exercise repetitions is comparable to a group of PTs. This supports potential to track patient progress through home physical therapy. Importantly, as video of each session was collected by the Kinect^™^ camera, the results support that Kinect^™^ video can be reliably used for patient assessment.

The VERA was used in a way similar to an actual clinical use case for home physical therapy: subjects were taught how to perform four different exercises by a PT, oriented to the VERA system, and then asked to perform the exercises unsupervised. Tools like the VERA could ensure the optimal dose of rehabilitation exercise is achieved by providing guidance to patients at home, while at the same time logging adherence and performance metrics for review. Presenting exercise metrics to patients and providers will likely promote increased patient engagement and adherence to the physical therapy process.

To our knowledge this is the first evaluation of an automated tele-rehabiliation system using the Microsoft Kinect^™^ camera to assess important rehabilitation metrics compared to experienced PTs. Across four diverse lower extremity exercises the VERA provided a repetition count with an acceptable level of accuracy compared to a seven member PT panel, underestimating the total count by less than 1%.

The data reflected the variance in seven PTs with similar backgrounds and practice settings assessed exercise “correctness.” The majority of the variance observed in the sample of raters was contributed not by the VERA but by PT1 and PT7 as these two raters did not exhibit consistency with the VERA or fellow PT raters. In practice, PT1 could be “strict” rater, rating fewer repetitions to be acceptable. In contrast, PT7 could be considered more lenient than the other raters, rating more repetitions as acceptable. These represent both ends of the spectrum, 1) a ‘normative’ cluster where most PTs converge, 2) a ‘strict’ outlier, and 3) a ‘lenient’ outlier.

To further explore the inter-rater variability, we performed a secondary chi square analysis after removing the more extreme raters PT1 and PT7. The results of the new chi square analysis were non-significant, = 5.53 (df = 4, p>0.1), indicating consistency and interchangeability with respect to the quality of repetition ratings produced by the VERA and PTs 2–6.

For the purpose of the VERA, the ability to capture the general parameters of acceptable was the goal. Results of the subgroup analysis including the VERA and PTs 2–6 supported a high degree of consistency in repetition quality assessment. Essentially, all raters in this group – the VERA and PTs 2–6 – represent a distinct cluster that can represent a standardized parameter of acceptable exercise in the manner in which they rated the quality of exercise repetitions.

Improper exercise form can be associated with teaching proper patterns of neuromuscular activation leading to greater injury. Additionally, lack of adherence to a structured home exercise program can result in delayed healing and prolonged return to functional activities or sports. In current practice methods to capture these metrics include patient self-report and periodic assessment in the physical therapy clinic. Unfortunately, these metrics are difficult to acquire in the home, where the majority of physical therapy takes place. Considering the high burden of functional disability and injury requiring rehabilitation, the supply-demand mismatch with physical therapy services, and high costs associated with prolonged physical therapy services, systems like the VERA may play an important role in providing cost-effective and efficient tele-rehabiliation services to patients at home.

## Limitations

There are several limitations to the present investigation. The consistency in how each group rated exercise repetitions was compared. An assessment of agreement between both groups per exercise and per exercise repetitions may be more appropriate using a larger sample of repetitions. Four lower extremity exercises were tested, additional exercises involving upper extremity joints and the use of exercise aids such as weights or resistance bands were not tested. Subjects in this study were healthy and without musculoskeletal injury. Future studies will include individuals with cognitive and physical impairments as well as those who are classified as obese to determine the feasibility of using the device on a wide range of body types and abilities. Additionally, unique patient characteristics such as demographics, injury type, and rehabilitation goals can influence assessment of exercise quality. This contextual information was not provided to the PTs as part of the study and may have altered the acceptability of repetitions. Finally, our protocol did not intentionally instruct subjects to perform repetitions with poor form resulting in the majority of repetitions characterized as acceptable. A larger number of intentionally unacceptable repetitions would have allowed for characterization of consistency between both the VERA and PT panel assessed unacceptable repetitions.

## Conclusion

The VERA was able to count exercise repetitions accurately in comparison to a group of PTs. The VERA tele-rehabilitation platform shows promise in serving as a clinically useful tool to collect important rehabilitation metrics for outpatient physical therapy without the need for PT supervision.

## Figures and Tables

**Table 1:** Total repetition count and number of acceptable vs. unacceptable repetitions by the VERA and each PT.

	VERA	PT1	PT2	PT3	PT4	PT5	PT6	PT7	Mean Counts (PTs)
Acceptable Repetitions	400	374	402	397	405	407	411	423	403 (SD = 15)
Unacceptable Repetitions	26	53	25	30	22	20	16	2	24 (SD = 16)
Total Repetitions	426	427	427	427	427	427	427	425	426.7 (SD = 0.8)

**Table 2:** Pair wise analysis between PTs and the VERA using Chi Square. Bold digits represent significant results.

	VERA	PT1	PT2	PT3	PT4	PT5	PT6	PT7
VERA	0							
PT1	10.10123311	0						
PT2	0.024595374	11.06159133	0					
PT3	0.297006632	7.059615895	0.485834566	0				
PT4	0.364389234	14.0469662	0.202641778	1.31056973	0			
PT5	0.843327407	16.31217442	0.586457904	2.124378109	0.100164204	0		
PT6	2.530150901	21.58452875	2.075240752	4.503443823	0.991486068	0.464004347	0	
PT7	21.21419892	50.30345614	20.12713805	25.32439024	17.05797101	15.03570646	11.06155076	0

**Table 3:** Chi square analysis of VERA and PT raters.

All Raters	63.17 (df=6, p<.001)
VERA and PTs 2–6	5.53 (df=4, p>0.1)
